# Врожденный гиперинсулинизм в составе синдрома Кабуки

**DOI:** 10.14341/probl13145

**Published:** 2022-07-20

**Authors:** А. Р. Бенина, М. А. Меликян

**Affiliations:** Национальный медицинский исследовательский центр эндокринологии; Национальный медицинский исследовательский центр эндокринологии

**Keywords:** синдром Кабуки, врожденный гиперинсулинизм, гипогликемия

## Abstract

Синдром Кабуки (СК) — редкое наследственное заболевание, характеризующееся стигмами дисэмбриогенеза, скелетными аномалиями, задержкой психомоторного развития, врожденными пороками развития. Врожденный гиперинсулинизм является редким проявлением СК, однако его ранняя диагностика крайне важна для предотвращения неврологических осложнений, связанных с гипогликемиями. Выделяют 2 типа СК, различающиеся по степени тяжести основных проявлений. СК 1-го типа ассоциирован с гетерозиготными мутациями в гене KMT2D. СК 2-го типа имеет Х-сцепленный тип наследования, является следствием гемизиготных мутаций в гене KDM6A и характеризуется более тяжелым течением заболевания. В данной статье мы приводим клиническое описание двух детей с врожденным гиперинсулинизмом в составе СК 1-го и 2-го типов.

## Abstract (en)

Kabuki syndrome is a rare hereditary disease characterized by distinctive facial features, skeletal abnormalities, mental retardation, developmental delay, and anomalies in multiple organ systems development.

Congenital hyperinsulinism is a rare manifestation of his Kabuki syndrome. However, early diagnosis is crucial to prevent neurological complications of hypoglycemia.

There are 2 types of Kabuki Syndrome depending on severity of symptoms. Kabuki syndrome Type 1 is associated with heterozygous mutations in gene KMT2D. Kabuki syndrome Type 2 is inherited in an X-linked manner. It’s associated with heterozygous mutations in gene KDM6A and characterized by more severe course of the disease.

This paper presents 2 cases of children with congenital hyperinsulinism as the feature of Kabuki syndrome Type 1 and Type 2.

## АКТУАЛЬНОСТЬ

Синдром Кабуки (СК) — это редкое наследственное заболевание, впервые описанное в 1981 г. Niikawa и соавт. и Kuroki и соавт. [1][2]. По данным литературы, распространенность синдрома составляет 1:32 000 в Японии и 1:86 000 в Австралии и Новой Зеландии [1]. Свое название данный синдром получил из-за схожести черт лица пациентов с маской японского театра Кабуки [3].

В настоящее время известно два гена, ответственных за развитие СК. Ген KMT2D расположен на 12 хромосоме и кодирует лизинспецифическую метилтрансферазу [1][4]. Гетерозиготные мутации в KMT2D приводят к развитию СК типа 1, наследуются аутосомно-доминантным путем и выявляются в 56–75% всех случаев СК [5].

Ген KDM6A расположен на Х-хромосоме и кодирует гистондеметилазу, которая воздействует на белок H3K27 и обеспечивает устойчивое состояние в пролиферирующих клетках [4][6]. Мутации в гене KDM6A приводят к развитию СК типа 2 и встречаются лишь у 5–8% пациентов с данным синдромом [2][ 5][7]. Гены KMT2D и KDM6A принимают участие в процессах эмбриогенеза [4]. Являясь модификаторами гистонов, участвуют в процессах соответствующей клеточной дифференцировки [4]. Несмотря на тип наследования, большинство мутаций, отвечающих за развитие СК, возникают de novo [6]. Этиология около 20% случаев СК остается неизвестной [5].

Выделяют 5 основных признаков СК: стигмы дисэмбриогенеза (длинные глазные щели с выворотом латеральной трети нижнего века, высокоизогнутые и широкие брови; короткая колумелла с вдавленным кончиком носа; большие, выпуклые и/или чашевидные уши); скелетные аномалии (аномалии позвоночника, клинодактилия/брахидактилия пятых пальцев); дерматоглифические аномалии (сохранение подушечек пальцев плода); задержка роста; умственная отсталость различной степени тяжести [8][9]. К редким проявлениям синдрома относятся врожденные пороки сердца, желудочно-кишечного тракта, мочеполовой системы, нарушения слуха, а также патологии эндокринной системы, которые включают в себя дефицит тропных гормонов гипофиза, надпочечниковую недостаточность, преждевременное половое развитие, изолированное телархе, врожденный гиперинсулинизм (ВГИ) [9].

ВГИ приводит к развитию тяжелых гипогликемий и требует особого внимания. В своих исследованиях авторы К. Lee Yap и соавт. отмечают, что на долю СК приходится около 1% всех случаев ВГИ, однако ввиду сложности в диагностике синдрома его истинная распространенность может быть выше [7].

В настоящее время патогенез гиперинсулинизма при СК неизвестен. В исследованиях M. Salguero и соавт. описана гипотеза, которая заключается в нарушении деметилирования белка H3K27 [6]. Во время дифференцировки клеток поджелудочной железы in vivo увеличивается количество доменов H3K27me3. Нарушение деметилирования метки H3K27me3/me2, связанной с геном KDM6A, приводит к избыточной пролиферации β-клеток, создавая основу для органического гиперинсулизма [6]. В своих работах H. Hoermann и K. Yap отмечают, что гиперинсулинемические гипогликемии чаще встречаются при СК типа 2 [7][10]. Очевидно, ген KDM6A в наибольшей степени оказывает влияние на работу β-клеток, чем KMT2D.

В большинстве случаев СК гиперинсулинизм имеет транзиторное течение. По данным литературы, пациенты с СК имеют хороший ответ на терапию диазоксидом, и лишь немногим требуется дополнительное лечение (аналоги соматостатина, хирургическое вмешательство) [7][10]. Учитывая высокий процент пороков сердца при СК, крайне необходимо проводить комплексную диагностику перед назначением терапии во избежание развития острой легочной гипертензии.

## ОПИСАНИЕ СЛУЧАЕВ

Были обследованы два ребенка с СК типов 1 и 2. Обоим пациентам диагноз был подтвержден молекулярно-генетически (табл. 1).

**Table:** Таблица 1. Клинико-диагностические особенности пациентов 1, 2Table 1. Clinical and diagnostic features of patients 1, 2 Примечание. ИФР1 — инсулиноподобный фактор роста 1-го типа; ЛГ — лютеинизирующий гормон; ФСГ — фолликулостимулирующий гормон; ТТГ — тиреотропный гормон; Т4св. — свободный тироксин; АКТГ — адренокортикотропный гормон.

Критерии	Пациент 1	Пациент 2
Синдром Кабуки	1 тип (мутации в KMT2D)	2 тип (мутации в KDM6A)
Мутация	Гетерозиготная мутация c.4843C>T:p.R1615X	Гемизиготная мутация c.2950 2954del:p.F984fs
Пол	Женский	Мужской
Гестационный возраст, нед	38	36
Длина тела при рождении, см	48	48
Масса тела при рождении, г	2780	2900
Возраст манифестации гипогликемий	1-е сутки жизни	5 мес
Уровень инсулина при манифестации	-	1,9 мкЕд/мл на фоне гипогликемии (2,6 ммоль/л)
Эффективная доза диазоксида	4,4 мг/кг/сут	-
Возраст ремиссии	3 года 4 мес	1 год 1 мес
Сопутствующие признаки	Стигмы дисэмбриогенеза:•брахидактилия;•широкие ступни и кисти;•крупный рот;•макроглоссия.Телархе.Снижение темпов роста.Задержка речевого развития	Стигмы дисэмбриогенеза:•длинный фильтр;•высокое готическое небо;•оттопыренные чашевидные ушные раковины;•двусторонний паховый крипторхизм.Задержка психомоторного развития
Гормональное исследование	ИФР1 — 252 нг/мл;ЛГ — 0,09 мМЕд/мл (1,7–8,6);ФСГ — 3,52 мМЕд/мл (1,5–12,4);эстрадиол — 3 пг/мл (6–27);ТТГ — 4,35 мМЕд/мл (0,7–5,97);Т4св. — 1,27 нг/дл (0,96–1,77)	ИФР 1 — 53,01 нг/мл;ЛГ — 0,216 Ед/л (0–1,5);ФСГ — 0,66 Ед/л (0–2);тестостерон — 0,48 моль/л (0,3–0,6);АКТГ — 81,68 пг/мл (7,2–63,3);кортизол — 690,6 нмоль/л (28–670);пролактин — 527,3 мЕд/л;ТТГ — 2,695 мМЕ/л (0,98–5,63);Т4св. — 12,68 пмоль/л (11,4–19,5)
Эхо-КГ	Отклонений не выявлено	Регистрируется открытое овальное окно, нельзя исключить впадение дополнительной верхней полой вены
УЗИ почек	Отклонений не выявлено	Отклонений не выявлено

## Пациент 1

Девочка родилась от второй нормально протекавшей беременности, первых родов, на 38-й неделе гестации. При рождении: масса тела — 2780 г, длина тела — 48 см. Эпизоды гипогликемий отмечались с первых дней жизни. При обследовании на 2-й неделе жизни установлен диагноз врожденного гиперинсулинизма. С 3 нед жизни назначена терапия диазоксидом в стартовой дозе 5 мг/кг/сут, с положительным эффектом. Стигм дисэмбриогенеза, врожденных пороков развития не отмечалось.

Для установления этиологии гиперинсулинизма проведено молекулярно-генетическое обследование (панель «Гиперинсулинизм» GCG, GLUD1, WFS1, HNF1A, GCK, INS, HNF1B, ABCC8, HNF4A, RFX6, PTF1A, NEUROD1, AKT2, ZFP57, INSR, EIF2AK3, PPARG, PAX4, PDX1, GLIS3, KCNJ11, SLC16A1, FOXP3, BLK, CEL, KLF11, SCHAD, GCGR, общее покрытие: 96,5%, методом NGS, параллельное секвенирование) — мутаций не выявлено.

Ребенок регулярно наблюдался эндокринологом. По результатам контрольных обследований отмечалась медикаментозная ремиссия заболевания. В 3 года 4 мес при очередном обследовании, учитывая низкую потребность в диазоксиде (2,7 мг/кг/сут), отсутствие гипогликемий за последний год, терапию было рекомендовано отменить. На фоне отмены препарата констатирована ремиссия ВГИ (стойкая эугликемия при проведении контрольной пробы с голоданием на фоне голодного промежутка в 8 ч, при гликемии 3,1 ммоль/л отмечалось подавление инсулина до 0,7 мкЕ/мл).

С первых месяцев жизни родители отмечали сниженный аппетит, частые срыгивания. Несмотря на сложность в кормлении, прибавка в весе была адекватной. С 1 года жизни отмечалось появление телархе, было выявлено повышение уровня эстрадиола до 126,7 пмоль/л (норма до 50 пмоль/л). По результатам УЗИ органов малого таза размеры матки и яичников превышали возрастную норму, выявлена киста правого яичника размером 0,8 см. По результатам обследования был диагностирован вариант изолированного телархе, что расценивалось как течение «мини-пубертата». Темпы психомоторного развития соответствовали возрасту.

С 1,5 года стали обращать на себя внимание стигмы дисэмбриогенеза (брахидактилия, широкие ступни и кисти, крупный рот, макроглоссия) (рис. 1 А, Б). Был заподозрен синдромальный вариант ВГИ. Проведено молекулярно-генетическое исследование — поиск аномального метилирования и дисомии генов IGF2 и LIT1, поиск делеций в регионе 7q11 — изменений выявлено не было.

**Figure:**
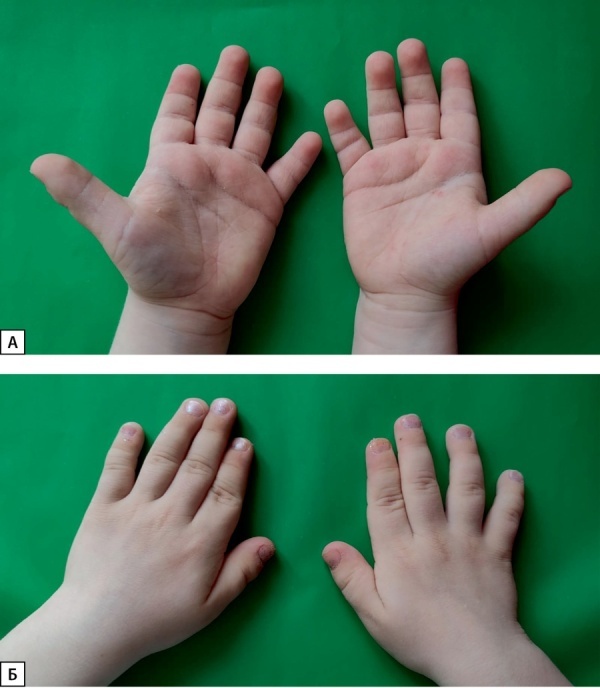
Рисунок 1. А — кисти пациента 1 (ладонная поверхность). Отмечаются брахидактилия, «плодные» подушечки пальцев, исчерченность ладоней. Б — кисти пациента 1.Figure 1. A — Patient 1's hands (palmar surface). Brachydactyly, "fetal" fingertips, striated palms are noted. B — hands of patient 1.

В возрасте 3 лет при очередном осмотре отмечено умеренное снижение темпов роста.

Учитывая наличие у ребенка транзиторного варианта ВГИ в сочетании со стигмами дисэмбриогенеза, было выполнено полное секвенирование экзома. По результатам исследования в гене KMT2D (NM 003482.4) в экзоне 20 выявлена гетерозиготная мутация c.4843C>T:p.R1615X (HGMD:CM105487; ClinVar:1012608), которая является патогенной и описана при СК типа 1 (OMIM #147920). После верификации диагноза девочке было проведено комплексное обследование на составляющие заболевания. По данным гормонального исследования отклонений не выявлено (табл. 1). По результатам Эхо-КГ и УЗИ почек отклонений не выявлено. Таким образом, из компонентов синдрома у ребенка имеются ВГИ, стигмы дисэмбриогенеза.

## Пациент 2

Мальчик родился от первой беременности, протекавшей на фоне угрозы прерывания в первой половине беременности, отслойки хориона на 5–6-й неделе, герпетической инфекции на 6–7-й неделе, тромбофилической коагулопатии на 33-й неделе (мать получала Клексан до 34 нед), хронической фетоплацентарной недостаточности, первых родов на 36-й неделе путем кесарева сечения. Масса тела при рождении 2900 г, длина тела 48 см, оценка по шкале Апгар 7/8 баллов.

В неонатальном периоде отмечались синдром угнетения, судорожный синдром, ранний неонатальный сепсис на фоне менингоэнцефалита. В течение недели находился на ИВЛ.

В связи со слабостью сосательного рефлекса до 1 мес жизни получал зондовое питание. В возрасте 2 мес жизни в связи с частыми срыгиваниями проведена фиброгастродуоденоскопия — выявлены физиологическая ахалазия, врожденный пилороспазм.

Ребенок наблюдался неврологом по поводу задержки психомоторного развития. В 5 мес при очередном обследовании впервые зафиксирована гипогликемия до 2,2 ммоль/л. На фоне голодного промежутка более 2 ч отмечались гипогликемии до 2,2–2,6 ммоль/л. Проведено дообследование: на фоне гликемии 2,6 ммоль/л уровень инсулина — 1,9 мкЕд/мл. Гипогликемии купировались частым дробным кормлением. Медикаментозную терапию не получал.

Был произведен анализ крови на спектр аминокислот и ацилкарнитинов методом тандемной масс-спектрометрии — патологии не выявлено. По данным молекулярно-генетического исследования методом NGS — анализ 587 генов, панель «Наследственные метаболические заболевания», — патологии не выявлено.

Впервые обследован эндокринологом в возрасте 7 мес. При осмотре обращали на себя внимание множественные стигмы дисэмбриогенеза: длинный фильтр, высокое готическое небо, оттопыренные чашевидные ушные раковины; двусторонний паховый крипторхизм; задержка психомоторного развития (ребенок держит голову с 7 мес, неуверенно, пытается поворачиваться с помощью, не сидит). По результатам пробы с голоданием был диагностирован ВГИ: на фоне голодного промежутка 8,5 ч уровень гликемии 2,7 ммоль/л, инсулин — 3,07 мкМЕ/мл, С-пептид — 1,04 нг/мл, кетоны — 0,3 ммоль/л. Назначена терапия диазоксидом в дозе 4 мг/кг/сут без эффекта. Также отмечалась непереносимость терапии в виде учащения срыгиваний,снижения аппетита, в связи с чем диазоксид был отменен. Эугликемии удалось добиться на фоне дробного режима кормления.

Учитывая наличие признаков дисморфогенеза в сочетании с гиперинсулинизмом, был заподозрен СК. Проведено молекулярно-генетическое исследование (полное экзомное секвенирование), по результатам которого в гене KDM6A (NM 021140.3) вэкзоне 20 выявлен гемизиготный вариант c.2950 2954del:p.F984fs. Данный вариант ранее в литературе не описан; расценен как патогенный. Мутации в гене KDM6A описаны при СК типа 2 (OMIM #300867). Было проведено комплексное обследование.По результатам гормонального профиля данных за наличие гипотиреоза, гипокортицизма, гипогонадизма не получено (табл. 1). При УЗИ почек патологии не выявлено. По данным Эхо-КГ регистрируется открытое овальное окно, нельзя исключить впадение дополнительной верхней полой вены.

На момент написания статьи ребенку 1 год 1 мес. Отмечаются жалобы на задержку роста, плохую прибавку веса, эпизоды диареи, задержку психомоторного развития. При осмотре рост 69 см, SDS роста -3,18; масса тела 7,9 кг, SDS ИМТ -0,59; микроцефалия: окружность головы 44 см, SDS окружности головы -2,04; задержка психомоторного развития (ребенок поворачивается со спины на живот, сидит с подмогой, голову держит неуверенно). Проведен анализ крови на антитела к глиадину итканевой трансглутаминазе для исключения целиакии как возможного компонента СК — результат отрицательный. Проведена коррекция питания. На фоне дробного режима питания гипогликемии не фиксируются, в то же время сохраняется высокий уровень инсулина (на фоне голодного промежутка 9 ч отмечается повышение уровня инсулина до 27 мкЕд/мл при уровне гликемии 3,6 ммоль/л без клинических проявлений).

## ОБСУЖДЕНИЕ

ВГИ — это гетерогенная группа заболеваний, характеризующихся неадекватной гиперпродукцией инсулина β-клетками поджелудочной железы, что приводит к тяжелой персистирующей гипогликемии. Причиной ВГИ чаще служат мутации генов, участвующих в регуляции секреции инсулина (KCNJ11, ABCC8, GLUD1, GCK и др.). Выделяют транзиторные варианты гиперинсулинизма, ассоциированные с осложнениями внутриутробного и перинатального периодов (задержка внутриутробного развития, асфиксия в родах, гестационный сахарный диабет и др.). Кроме того, описан целый ряд синдромальных патологий, в структуру которых может входить ВГИ. СК — одна из редких причин ВГИ.

СК — это многокомпонентное заболевание, характеризующееся типичными стигмами дисэмбриогенеза, врожденными аномалиями развития, задержкой роста и психомоторного развития. В настоящее время известно два гена, отвечающих за развитие синдрома Кабуки типов 1 и 2 соответственно: KMT2D и KDM6A. Гиперинсулинизм является редким проявлением синдрома, однако его ранняя диагностика крайне важна для предотвращения нейрогликопенических осложнений ЦНС. Характер течения гипогликемии при СК может быть транзиторным или перманентным [11]. Препаратом первой линии для лечения гиперинсулинизма является диазоксид. Нами были обследованы два ребенка с СК типов 1 и 2 (табл. 1).

У пациента 1 признаки гиперинсулинизма манифестировали с 1-х суток жизни и регрессировали в возрасте 3 лет. Учитывая отсутствие типичных стигм дисэмбриогенеза в младенческом возрасте, постановка диагноза СК была затруднена. Течение ВГИ расценивалось как проявление перинатального стресса, в пользу чего говорили некрупные вес и рост при рождении. Характерные для СК черты лица, снижение темпов роста и явления телархе были отмечены уже после года жизни. Известно, что степень выраженности фенотипических проявлений СК увеличивается по мере взросления ребенка, в связи с чем заболевание диагностируется в более поздний период [4][11]. Авторы A. Subbarayan и K. Hussain описали пациентов с гетерозиготной мутацией в гене MLL2 (прежнее название гена KMT2D), чей диагноз был установлен в возрасте от 1 года и старше [11].

У пациента 2 гиперинсулинизм установлен в возрасте 5 мес. Ребенок имел типичные стигмы дисэмбриогенеза (рис. 2А, Б), задержку психомоторного развития, крипторхизм (табл. 1). Мутация у данного пациента ранее не описана в литературе, и, вероятно, бессимптомное течение гиперинсулинизма является особенностью ее клинического проявления. Однако стоит отметить, что, по данным исследования K. Yap и соавт., риск возникновения неонатальной гипогликемии при мутации в генеKDM6A выше, чем при мутациях в KMT2D [7][10]. В нашем случае наблюдается обратная ситуация, что, вероятнее всего, носит характер исключения.

**Figure:**
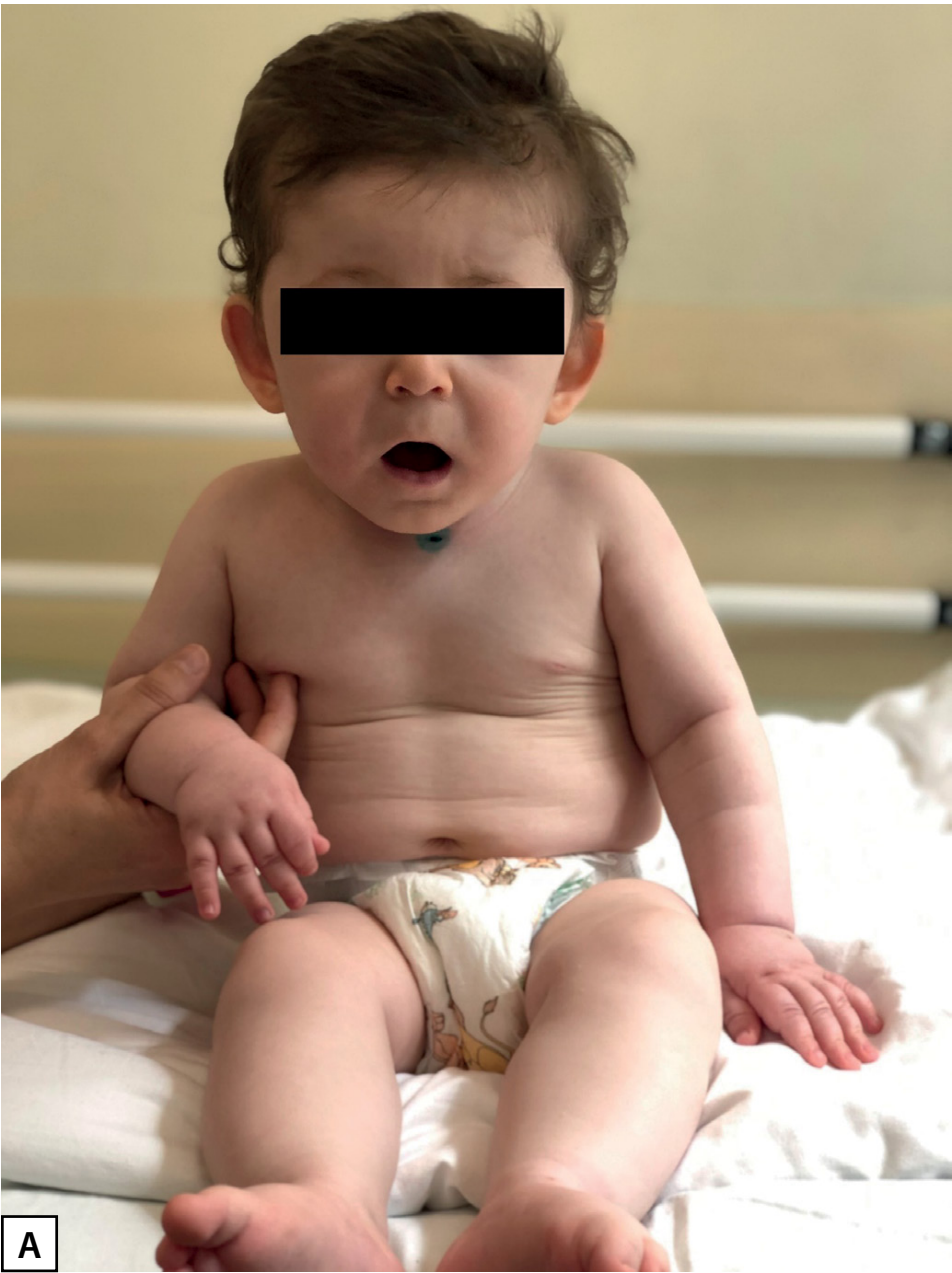
Рисунок 2А. Фото пациента 2. Стигмы дисэмбриогенеза: длинный фильтр, оттопыренные чашевидные ушные раковины.Figure 2A. Photo of the patient 2. Dysembryogenesis stigmas: long filter, protruding cupped auricles.

**Figure:**
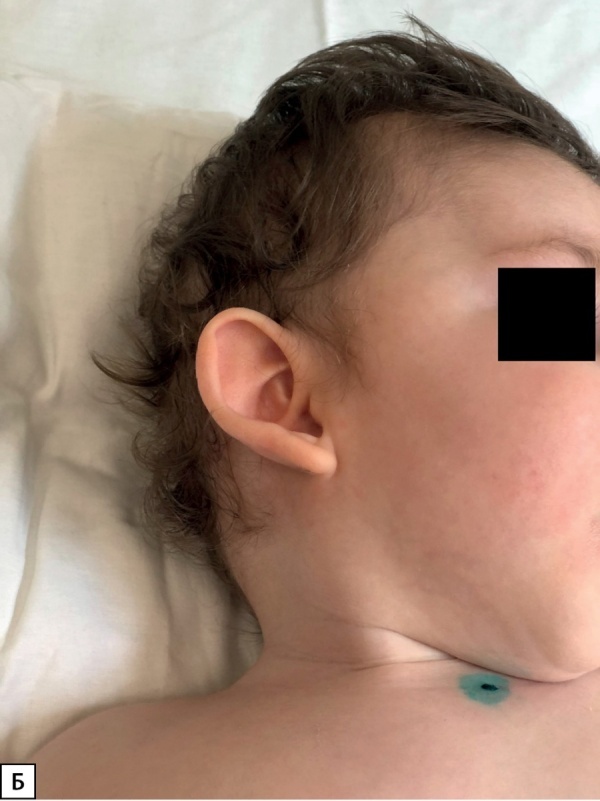
Рисунок 2Б. Фото пациента 2. Стигмы дисэмбриогенеза: оттопыренные чашевидные ушные раковины.Figure 2B. Photo of the patient 2. Stigmas of disembryogenesis: protruding cup-shaped auricles.

У обследованных нами детей гипогликемия носила транзиторный характер, и один из них имел хороший ответ на терапию диазоксидом, что характерно для течения СК. Вопрос об отсутствии клинической картины гипогликемии на фоне высокой секреции инсулина у нашего пациента остается открытым, поскольку патогенез развития гиперинсулинизма при СК является малоизученным.

При диагностировании СК необходимо проводить скрининг на ВГИ, поскольку гиперинсулинизм, как в случае с нашим пациентом, может иметь бессимптомное или малосимптомное течение. И наоборот, при выявлении синдромального органического гиперинсулинизма необходимо проводить исследование на составляющие СК для своевременной диагностики осложнений, связанных с терапией ВГИ.

## ЗАКЛЮЧЕНИЕ

Ввиду неярко выраженных признаков дисморфогенеза СК гипогликемия у новорожденных может быть расценена как транзиторная. Для лечения гиперинсулинизма препаратом первой линии является диазоксид. Ранняя диагностика СК крайне важна не только для выявления гиперинсулинизма, но и для диагностики сопутствующих патологий. При проведении молекулярно-генетического анализа на выявление причин гипогликемий следует также исследовать мутации в генах KMT2D, KDM6A.

## ДОПОЛНИТЕЛЬНАЯ ИНФОРМАЦИЯ

Источники финансирования. Работа выполнена по инициативе авторов без привлечения финансирования.

Конфликт интересов. Авторы декларируют отсутствие явных и потенциальных конфликтов интересов, связанных с содержанием настоящей статьи.

Участие авторов. Все авторы одобрили финальную версию статьи перед публикацией, выразили согласие нести ответственность за все аспекты работы, подразумевающую надлежащее изучение и решение вопросов, связанных с точностью или добросовестностью любой части работы.

Согласие пациентов. Пациенты добровольно подписали информированное согласие на публикацию персональной медицинской информации в обезличенной форме в журнале «Проблемы эндокринологии».

## References

[cit1] Mısırlıgil Mina, Yıldız Yılmaz, Akın Onur, Odabaşı Güneş Sevinç, Arslan Mutluay, Ünay Bülent (2020). A Rare Cause of Hyperinsulinemic Hypoglycemia: Kabuki Syndrome. Journal of Clinical Research in Pediatric Endocrinology.

[cit2] Bögershausen N, Wollnik B (2012). Unmasking Kabuki syndrome. Clinical Genetics.

[cit3] “Kabuki Syndrome Clinical Management Guidelines Management of Kabuki Syndrome A Clinical Guideline.”

[cit4] Gole Hobia, Chuk Raymond, Coman David (2016). Persistent Hyperinsulinism in Kabuki Syndrome 2: Case Report and Literature Review. Clinics and Practice.

[cit5] Boniel Snir, Szymańska Krystyna, Śmigiel Robert, Szczałuba Krzysztof (2021). Kabuki Syndrome—Clinical Review with Molecular Aspects. Genes.

[cit6] Salguero Maria V, Chan Karen, Greeley Siri Atma W, Dyamenahalli Umesh, Waggoner Darrel, del Gaudio Daniela, Rajiyah Tara, Lemelman Michelle (2022). Novel KDM6A Kabuki Syndrome Mutation With Hyperinsulinemic Hypoglycemia and Pulmonary Hypertension Requiring ECMO. Journal of the Endocrine Society.

[cit7] Yap Kai Lee, Johnson Amy E. Knight, Fischer David, Kandikatla Priscilla, Deml Jacea, Nelakuditi Viswateja, Halbach Sara, Jeha George S., Burrage Lindsay C., Bodamer Olaf, Benavides Valeria C., Lewis Andrea M., Ellard Sian, Shah Pratik, Cody Declan, Diaz Alejandro, Devarajan Aishwarya, Truong Lisa, Greeley Siri Atma W., De Leó-Crutchlow Diva D., Edmondson Andrew C., Das Soma, Thornton Paul, Waggoner Darrel, del Gaudio Daniela (2018). Congenital hyperinsulinism as the presenting feature of Kabuki syndrome: clinical and molecular characterization of 10 affected individuals. Genetics in Medicine.

[cit8] Zarate Y.A., Zhan H., Jones J.R. (2012). Infrequent Manifestations of Kabuki Syndrome in a Patient with Novel <b><i>MLL2</i></b> Mutation. Molecular Syndromology.

[cit9] Adam MP, Hudgins L, Syndrome HMK, et al. Kabuki Syndrome Synonyms: Kabuki Make-Up Syndrome, Niikawa-Kuroki Syndrome. GeneReviews. 2011.

[cit10] Hoermann Henrike, El‐Rifai Omar, Schebek Martin, Lodefalk Maria, Brusgaard Klaus, Bachmann Nadine, Bergmann Carsten, Roeper Marcia, Welters Alena, Salimi Dafsari Roschan, Blankenstein Oliver, Mayatepek Ertan, Christesen Henrik, Meissner Thomas, Kummer Sebastian (2020). Comparative meta‐analysis of Kabuki syndrome with and without hyperinsulinaemic hypoglycaemia. Clinical Endocrinology.

[cit11] Subbarayan Anbezhil, Hussain Khalid (2013). Hypoglycemia in Kabuki syndrome. American Journal of Medical Genetics Part A.

